# 1143. Prophylactic and Therapeutic Activity of AZD7442 (Tixagevimab/Cilgavimab) in SARS-CoV-2 Hamster Challenge Models

**DOI:** 10.1093/ofid/ofac492.981

**Published:** 2022-12-15

**Authors:** Yueh-Ming Loo, Andrew S Herbert, Ana I Kuehne, Patrick M McTamney, Richard Roque, Alicia M Moreau, Russell Bakken, Christopher P Stefan, Jeffrey W Koehler, Korey L Delp, Susan R Coyne, Christopher D Kane, John M Dye, Yingyun Cai, Mark T Esser

**Affiliations:** AstraZeneca, Gaithersburg, Maryland; United States Army Medical Research Institute of Infectious Diseases, Fort Detrick, Maryland; United States Army Medical Research Institute of Infectious Disease, Fort Detrick, Maryland; AstraZeneca, Gaithersburg, Maryland; AstraZeneca, Gaithersburg, Maryland; United States Army Medical Research Institute of Infectious Diseases, Fort Detrick, Maryland; United States Army Medical Research Institute of Infectious Diseases, Fort Detrick, Maryland; United States Army Medical Research Institute of Infectious Diseases, Fort Detrick, Maryland; United States Army Medical Research Institute of Infectious Diseases, Fort Detrick, Maryland; United States Army Medical Research Institute of Infectious Diseases, Fort Detrick, Maryland; United States Army Medical Research Institute of Infectious Diseases, Fort Detrick, Maryland; United States Army Medical Research Institute of Infectious Diseases, Fort Detrick, Maryland; United States Army Medical Research Institute of Infectious Diseases, Fort Detrick, Maryland; AstraZeneca, Gaithersburg, Maryland; AstraZeneca, Gaithersburg, Maryland

## Abstract

**Background:**

AZD7442—a combination of 2 human, extended–half-life, SARS-CoV-2–neutralizing monoclonal antibodies (mAbs) (tixagevimab/cilgavimab)—has received US Food and Drug Administration emergency use authorization for COVID-19 prevention in immunocompromised individuals. We evaluated the effect of AZD7442 in prevention and treatment settings in Syrian hamsters challenged with SARS-CoV-2.

**Methods:**

Hamsters received intraperitoneal isotype control mAb (2 mg) or AZD7442 (0.002–2 mg) 1 day before intranasal (IN) SARS-CoV-2 challenge (USA-WA1/2020; 1x10^5^ plaque-forming units) in prevention; OR control mAb (5 mg) or AZD7442 (0.5–5 mg) 1 day after IN SARS-CoV-2 challenge in treatment. The impact of AZD7442 on lung viral RNA and pathology and AZD7442 serum levels was assessed on Days 3 and 7 post infection. Body weight was recorded daily through Day 7.

**Results:**

With AZD7442 prevention, lower lung viral loads were observed compared to controls; at Day 3 post infection, lowest infectious virus titer and viral subgenomic mRNA (sgmRNA) levels were seen with doses ≥0.2 mg AZD7442. Concomitantly, increased serum levels of AZD7442 were observed. By Day 7, infectious virus titer and sgmRNA fell below the level of detection (LOD) at all doses tested. Moreover, AZD7442 at doses ≥0.2 mg protected hamsters from weight loss versus controls. Lung pathology scores (scale: 0 [normal] to 25 [most severe]) were generally dose dependent, with mean scores of < 2 for AZD7442 versus 10 for controls, indicating less SARS-CoV-2–induced inflammation and alveolar damage in hamsters given AZD7442. Lower AZD7442 doses were associated with mean pathology scores similar to controls. With AZD7442 treatment, infectious virus titers were below the LOD at Day 3 post infection and at Day 7 for sgmRNA, for all doses tested. Mean lung pathology score was <2 for AZD7442 versus 12 for controls. AZD7442 doses ≥0.5 mg protected against weight loss relative to controls.

**Figure:**
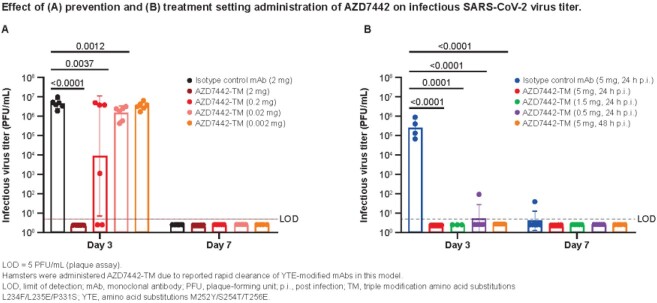

**Conclusion:**

In a SARS-CoV-2 challenge model, AZD7442 administered as prevention or treatment led to significantly lower lung viral loads and improved lung pathology, without weight loss. There was also no evidence that AZD7442 mediated antibody-dependent enhancement of disease or infection.

**Disclosures:**

**Yueh-Ming Loo, PhD**, AstraZeneca: Employee|AstraZeneca: Stocks/Bonds **Patrick M. McTamney, PhD**, AstraZeneca: Employee **Richard Roque, B.S.**, AstraZeneca: Employee|AstraZeneca: Stocks/Bonds **Yingyun Cai, PhD**, AstraZeneca: Employee|AstraZeneca: Stocks/Bonds **Mark T. Esser, PhD**, AstraZeneca: Employee|AstraZeneca: Stocks/Bonds.

